# Distinct phenotypes of cancer cells on tissue matrix gel

**DOI:** 10.1186/s13058-020-01321-7

**Published:** 2020-07-31

**Authors:** Kelsey F. Ruud, William C. Hiscox, Ilhan Yu, Roland K. Chen, Weimin Li

**Affiliations:** 1 Department of Biomedical Sciences, Elson S. Floyd College of Medicine, Washington State University, Spokane, WA 99202 USA; 2 Center for NMR Spectroscopy, Washington State University, Pullman, WA 99164 USA; 3 School of Mechanical and Materials Engineering, Washington State University, Pullman, WA 99164 USA

**Keywords:** Breast cancer, Extracellular matrix (ECM), Hydrogel, Tissue matrix gel (TMG), Receptor, Differentiation, Migration and invasion, Metabolism, Proliferation

## Abstract

**Background:**

Breast cancer cells invading the connective tissues outside the mammary lobule or duct immerse in a reservoir of extracellular matrix (ECM) that is structurally and biochemically distinct from that of their site of origin. The ECM is a spatial network of matrix proteins, which not only provide physical support but also serve as bioactive ligands to the cells. It becomes evident that the dimensional, mechanical, structural, and biochemical properties of ECM are all essential mediators of many cellular functions. To better understand breast cancer development and cancer cell biology in native tissue environment, various tissue-mimicking culture models such as hydrogel have been developed. Collagen I (Col I) and Matrigel are the most common hydrogels used in cancer research and have opened opportunities for addressing biological questions beyond the two-dimensional (2D) cell cultures. Yet, it remains unclear whether these broadly used hydrogels can recapitulate the environmental properties of tissue ECM, and whether breast cancer cells grown on CoI I or Matrigel display similar phenotypes as they would on their native ECM.

**Methods:**

We investigated mammary epithelial cell phenotypes and metabolic profiles on animal breast ECM-derived tissue matrix gel (TMG), Col I, and Matrigel. Atomic force microscopy (AFM), fluorescence microscopy, acini formation assay, differentiation experiments, spatial migration/invasion assays, proliferation assay, and nuclear magnetic resonance (NMR) spectroscopy were used to examine biological phenotypes and metabolic changes. Student’s *t* test was applied for statistical analyses.

**Results:**

Our data showed that under a similar physiological stiffness, the three types of hydrogels exhibited distinct microstructures. Breast cancer cells grown on TMG displayed quite different morphologies, surface receptor expression, differentiation status, migration and invasion, and metabolic profiles compared to those cultured on Col I and Matrigel. Depleting lactate produced by glycolytic metabolism of cancer cells abolished the cell proliferation promoted by the non-tissue-specific hydrogel.

**Conclusion:**

The full ECM protein-based hydrogel system may serve as a biologically relevant model system to study tissue- and disease-specific pathological questions. This work provides insights into tissue matrix regulation of cancer cell biomarker expression and identification of novel therapeutic targets for the treatment of human cancers based on tissue-specific disease modeling.

## Background

The extracellular matrix (ECM) of connective tissues is a spatial protein network that provides structural and biochemical essence for the survival and biological functions of tissue cells. ECM is made up of various fibrous proteins such as collagens, elastins, fibronectins, laminins, and proteoglycans and can have varying degrees of elasticity, stiffness, density, and protein composition that is often tissue-specific [1–4]. These existing yet invisible physicochemical properties affecting cellular signaling and response are mostly absent in two-dimensional (2D) tissue cultures and compromised in non-tissue-specific culture systems.

Since cell surface receptor expression [5] and numerous cellular functions are regulated by cell-ECM interactions [6, 7], alterations in the structures or compositions of tissue microenvironment around cells can affect their biological behaviors and pathological progression [8]. For example, the transition of ductal carcinoma in situ (DCIS) to invasive ductal carcinoma (IDC) is potentially regulated by alterations in epithelial-stromal interactions, changes in stromal cell gene expression, and matrix metalloproteinase-mediated tissue remodeling [9, 10]. On the other hand, tissue ECM modification can also be a natural process. For instance, normal stem cells may modify their surrounding ECM for better biological functions [11–14]. Interestingly, it was shown that normalizing tumor ECM environment was able to “revert” cancer cell neoplastic phenotype [15] and limit tumor growth and dissemination [16], highlighting the essential role of ECM physicochemical properties on cancer development. These ECM-guided cell biological changes suggest potential therapeutic approaches that target ECM rather than cancer cells for treatment or that treatments can be ineffective if, for example, a dense ECM or binding with the ECM limits drug availability [10, 17, 18]. Therefore, it is important to study cancer cell biology and regulatory mechanisms in tissue-mimicking microenvironments, which are able to facilitate unveiling the nature of carcinogenic processes when it comes to cancer cell adhesion, proliferation, migration, invasion, metastasis, and drug treatment in native tissue space [19–21].

Hydrogel is one of the most popular formats of biomaterials used for tissue mimicry in research [22]. Yet, the large number of different molecules that constitute ECMs of different tissues (thirty-plus different collagen types, twenty other proteins with collagen-like domains, more than thirty proteoglycans, fifteen different laminins, etc. [1, 8]) renders challenges for producing a universal hydrogel to accurately mimic individual tissue microenvironments. As Collagen I (Col I) is the most abundant ECM protein in the majority of mammalian connective tissues [23], with exceptions in a couple of tissues such as cartilage (mainly Col II) [24] and basement membrane (Col IV dominates) [25], it has been widely used as an ECM substitute in cell cultures. The major drawbacks of using Col I as a substratum for tissue cultures are the lack of the structural and biochemical heterogeneities of tissue ECM, requirement of chemical crosslinking for higher stiffness (> 1 kPa), limited long-term stability [26, 27], and temperature-sensitive variations in fibril formation [28], etc. Nevertheless, collagen is a valuable biomaterial for the observations of spatial cell migration [29, 30] and invasion [31, 32] as well as for bioengineering applications [33, 34].

Another native ECM-derived hydrogel commonly used in cancer research is Matrigel (also known as Cultrex BME or lrECM). Matrigel is extracted from Engelbreth-Holm-Swarm (EHS) mouse tumor whose origin is not well defined [35–37]. It has been used as a basement membrane alternative in tissue cultures. Normal and malignant mammary epithelial cells grown on Matrigel exhibit distinct colonized structures with different polarization and proliferation phenotypes [38]. Matrigel mainly contains about 60% laminin, 25–30% Col IV, perlecan and nidogen, with Col I at about 5%. Besides the homogeneous, nonfibrillar characteristics [36] and flipped Col IV and laminin ratios compared to native basement membrane compositions [25], the different growth factors that can be many folds above serum levels (even after growth factor reduction) and some underdefined constituents inherent to the EHS tumor confine the biological research applications of Matrigel as well as the interpretations for the phenotypes and mechanisms therein. However, Matrigel has been instrumental in many aspects of our understanding about 3D tumor biology and remains as a useful tool for basement membrane- or laminin/Col IV-related studies and maintenance of certain stem cells.

To understand breast cancer cell biology at tissue-specific and native environment-mimicry levels, we have recently developed mouse and porcine breast ECM hydrogels, termed tissue matrix gel (TMG), and its derived porous scaffold [39, 40]. TMG contains all the proteins found in natural breast ECM, including different types of collagens, laminins, fibronectin, other glycoproteins, and proteoglycans. The diverse ECM protein ligands and their natural abundances in TMG relative to Col I and Matrigel implicate distinct biological activities of the cells living on TMG compared to those on the latter two matrices. Importantly, the tissue- and disease-specific aspects of cell culture substrata are often considered essential elements for the biological relevance of 3D cultures [41, 42]. Here we present data to show the microstructural differences of TMG, Col I, and Matrigel and the features of mammary epithelial cell morphology, surface biomarker expression, differentiation, migration, invasion, and metabolism when they were grown on the three types of hydrogels. These characterizations provide valuable references for breast cancer research based on the use of native ECM gels.

## Methods

### Hydrogel

Three types of native tissue-derived hydrogels, TMG, Col I, and Matrigel, at physiological stiffness were used to study and compare the phenotypes and metabolic profiles of human mammary epithelial cells grown on the matrices. TMG was extracted from porcine breast tissue ECM as we previously reported [40]. Briefly, fresh mammary tissues from female pigs were collected from a local slaughterhouse, homogenized, decellularized, ECM total protein extracted, and reconstituted into hydrogel at desired concentrations for the experiments. Human Col I and Matrigel (GFR) were from Advanced BioMatrix and Corning, respectively.

### Analysis and statistics

Experimental comparisons, statistical analyses, and instrument-specific analytical methods were described in the respective sections within the “Methods” section. A statistical significance was defined as a *P* value less than 0.05, as indicated in the respective figure legends.

### Atomic force microscopy

AFM (Dimension Icon ScanAsyst, Santa Barbara, CA, USA) was used to determine Young’s moduli of the samples. The different types of hydrogels at the indicated concentrations were coated (1 cm in diameter, 20 μm thick) on Silane-Prep slides, polymerized at 37 °C for 1 h, and measured following the procedures we previously reported [40]. Borosilicated spherical-shaped probe tips at 5 μm diameter and AFM cantilever spring constant of 0.06 N/m were used in this study. The AFM tests were performed with a total indentation depth of 1 μm and an indentation rate of 5 μm/s. At least seven data points were collected from randomly selected regions from each sample. Hertz’s model was used to calculate Young’s modulus.

### Cell culture

MDA-MB-231 (MM231), MDA-MB-468 (MM468), T47D, BT474, and SKBR3 breast cancer cells and MCF10A normal human mammary epithelial cells were from ATCC; normal primary human mammary epithelial cells (HUMEC) were from ScienCell. The cancer cells were grown in 1× Dulbecco’s modification of Eagle’s medium (DMEM, Corning) supplemented with 10% fetal bovine serum (FBS) and 1% penicillin/streptomycin (Gibco) at 37 °C (5% CO_2_). The HUMEC cells were grown in Mammary Epithelial Cell Medium (ScienceCell). The MCF10A cells were grown in DMEM/F12 (Corning) supplemented with 5% horse serum, 20 ng/ml EGF, 1% penicillin/streptomycin, 0.5 μg/ml hydrocortisone, 100 ng/ml cholera toxin, and 10 μg/ml human insulin.

### Immunofluorescence (IF) staining and microscopy

Silicone wells attached to Silane-Prep slides (Sigma) were coated with 10 μl of TMG (2 mg/ml), human Col I (2 mg/ml, Advanced Biomatrix), or Matrigel (5 mg/ml, Corning). The gels were polymerized in a 37 °C incubator for 1 h. MM231 cells were seeded on the matrix (2000 cells/well) and cultured for indicated hours (37 °C, 5% CO_2_). The cells were then fixed with 4% paraformaldehyde, washed with 1× PBS, permeabilized with 0.1% Triton-X 100, blocked with 3% BSA in PBS, and incubated with Col I antibody (Abcam) and Alexa Fluor probe-conjugated phalloidin (ThermoFisher) in dark at 4 °C overnight. The cells were washed with 0.1% Triton-X 100 and incubated with Alexa Fluor 488-conjugated secondary antibody (ThermoFisher) against Col I for 1 h in dark at room temperature, followed by washing with 0.1% Triton-X 100 and Hoechst (TOCRIS) staining of cell nucleus. Coverslips were mounted on top of the IF-stained samples using FluorSave medium (MilliporeSigma). The slides were stored in dark at 4 °C overnight and staining imaged using a Zeiss Imager M2 upright epifluorescence microscope. The protrusions of 10 randomly selected cells cultured on individual matrix-coated slides were counted, and the collective counts from three biological repeats were compiled and compared using Student’s *t* test.

### Acini formation

Chamber well slides were coated with 40 μl of TMG (2 mg/ml) or Matrigel (5 mg/ml), and the gels were polymerized in a 37 °C incubator. MCF10A cells were mixed into 400 μl of culture medium containing 2% hydrogel (4000 cells/well), seeded on top of the polymerized gel, and cultured for 14 days. The cells were probed with primary antibodies against β1 or β4 integrin, and E-cadherin (Santa Cruz Biotechnology), followed by fluorophore-conjugated secondary antibody and Hoechst staining and imaged for acini structures under a fluorescence microscope.

### Differentiation assay

The wells of 8-well chamber slides were coated with 40 μl of TMG (2 mg/ml), Col I (2 mg/ml), or Matrigel (5 mg/ml), and the gels were polymerized in a 37 °C incubator. MM231, MM468, T47D, BT474, or SKBR3 cells were suspended in 400 μl of 1× DMEM containing 2% hydrogel, seeded on top of the polymerized gel (6000 cells/well), and cultured for 5–7 days. The cells were probed with primary antibodies against E-cadherin (Santa Cruz Biotechnology), Vimentin (Novus Biologicals), and ZO-1 (Thermo Fisher Scientific), followed by fluorophore-conjugated secondary antibody and Hoechst staining, and imaged under a fluorescence microscope.

### Cell migration assay

The wells of 24-well plates were coated with the different types of hydrogels (the same concentrations as used in the IF staining; 50 μl/well), which were polymerized at 37 °C for 2 h. Circular spacer wells were placed individually to the center of the coated wells. GFP-MM231 cells (6000 cells/well in 100 μl of 1× DMEM) were added to the spacer wells. The spacers were removed (1 h after cell seeding) after the cells were attached to the matrices. An additional 400 μl of medium per culture well was added after spacer removal. Cell migration from the center of the well outward on the hydrogel was imaged using a Zeiss inverted fluorescence microscope at 0, 24, 48, and 72 h. Three biological repeats were performed for statistical analysis using Student’s *t* test.

### Cell invasion assay

A piece of circular Nytran N Nylon membrane (pre-wetted with 1× PBS) was placed at the bottom of a silicone well on a Silane-Prep slide and 50 μl of each matrix (same concentrations as used in the assays described above) coated on top. The gels were polymerized in a 37 °C incubator for 2 h. GFP-MM231 cells were seeded on top of the matrix (2500 cells/well in 120 μl 1× DMEM) and cultured for 24 h. The cells and matrix were washed twice with cold 1× PBS and fixed with 4% paraformaldehyde at room temperature for 30 min. The hydrogel was gently scraped off, and the membrane rinsed with 1× PBS and imaged (2.5× magnification) under a Zeiss Imager M2 epifluorescence microscope. Alternatively, the membrane could be stained with Giemsa dye overnight, rinsed with 50% ethanol and 1× PBS, and imaged under a light microscope. Three biological repeats were performed for statistical analysis using Student’s *t* test.

### ^1^H HRMAS nuclear magnetic resonance (NMR) spectroscopy

HUMEC or MM231 cells were seeded on polymerized TMG (2 mg/ml), Col I (2 mg/ml), or Matrigel (5 mg/ml) in triplicates within 96-well plates (4000 cells/well), supplemented with 1× RPMI 1640 medium (Corning) containing 1 g/l of ^13^C_6_-labeled d-glucose (Cambridge Isotope Laboratories) and cultured under optimal conditions (37 °C, 5% CO_2_) for up to 7 days. The media of the cultures were collected for NMR analysis. The medium-only and 1-h culture samples were used as background controls.

High-resolution magic angle spinning (HRMAS) NMR spectroscopy was performed on a Varian VNMRS 500MHz NMR spectrometer equipped with an Agilent HRMAS probe (^1^H-observe, X-tunable), where the X-decoupling channel was tuned to ^13^C. HRMAS rotors (Agilent, glass, 30 μl) were used for all acquisitions. Five microliters of trimethylsilylpropanoic acid sodium salt in D_2_O (TMSP, 1.15 mg/ml) stock solution was transferred into the rotor using a 10.0-μl syringe. A calibrated pipette with a modified sampling tip was used to transfer a reproducible amount (24.0 μl) of the medium sample into the 30-μl rotor. A small void space or bubble (approximately 1 μl) was allowed between the sample and the bottom of the sealing piston of the rotor. Sample rotors were mounted in the stator of the probe and spun continuously at 2500 Hz at the magic angle (54.7°) for all of the samples. The temperature was equilibrated to 25 °C. Proton spectra were collected with presaturation of the water signal at 4.82 ppm (relative to TMSP SiMe_3_ at 0.0 ppm) with a saturation delay of 2 s (total recycle delay of 5 s), saturation power of 24 dB, acquiring 512 scans for each acquisition, with a calibrated 90° pulse equal to 7.5 μs, spectral width of 8012.8 Hz, acquisition time 0.750 s, collecting 6010 complex points.

Two sets of ^1^H NMR data were collected for each sample, one with no ^13^C decoupling (decoupler off), and the complimentary but opposite data set employing ^13^C-decoupling during acquisition (inverse gated decoupling) in order to distinguish incorporation of ^13^C from labeled glucose into other metabolites during the incubation period. Fully ^13^C-decoupled ^1^H NMR spectra represent hydrogen atoms bonded to all carbon isotopes, showing only ^1^H-^1^H scalar coupling, and masking ^13^C-^1^H coupling of protons bonded to ^13^C isotopes. Non-decoupled proton spectra of natural abundance carbon (consisting of 1.1% NMR active ^13^C) show a central peak for protons bonded to ^12^C, and peak splitting due to scalar coupling of ^13^C to the bonded protons. These so-called ^13^C satellites have an amplitude 0.55% of the center peak, based on total natural abundance ^13^C. Data were processed using VNMRJ 4.2 software. 2.0 Hz of line broadening was applied to each of the free induction decay (FID) data sets, which were Fourier transformed to yield the frequency spectra. Spectra were generally referenced to TMSP at 0.0 ppm. Selected assignments are based on previous works elsewhere [43–45].

### Proliferation assay

Flat bottom 96-well plates were coated with 5 μl of TMG (2 mg/ml), Col I (2 mg/ml), or Matrigel (5 mg/ml) in the presence or absence of treatment supplements where necessary. After gel polymerization for 1 h at 37 °C, HUMEC, MM231, MM468, T47D, BT474, or SKBR3 cells were seeded into the wells (1000 cells/well) and cultured for 5 days. Non-coated wells with medium and coated wells with medium were included as background controls. Each experimental condition was in triplicate. Cell proliferation was assessed using the WST-1 reagent (Roche) and absorbance reading over a Synergy microplate reader (BioTek) following the manufacturers’ instructions. Three biological repeats were performed for statistical analysis using Student’s *t* test.

## Results

### The mechanical and structural properties of the biogels with native tissue stiffness

The stiffness of a hydrogel is a critical factor for cell adhesion [46], spreading [47], proliferation [48, 49], migration [46, 50], invasion [51, 52], differentiation [53], and other biological activities such as neodeposition of matrix proteins [54], interaction with surrounding matrix or cells [55], and expression of gene products [56]. Cells originated from different tissues may require quite different substrate stiffness for optimal survival and growth [57]. On the other hand, cells living on a matrix can modify the mechanical properties of the matrix depending on the cellular and matrix conditions [58]. It is therefore important to keep the stiffness of hydrogel in the same range of the corresponding tissues to be mimicked when studying the biology of the cells living in those tissues.

**Fig. 1:**
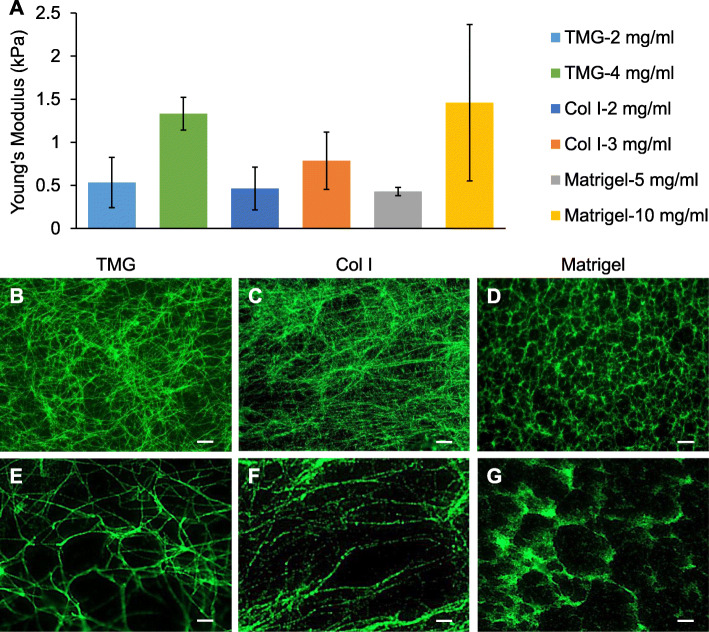
The elasticity and microstructures of the three types of hydrogels. **a** AFM measurement of hydrogel stiffness expressed using Young’s modulus. Two concentrations of the three types of hydrogels were used to representatively demonstrate the differences or similarities of the mechanical property between the gels. **b**–**d** The microstructures of the polymerized TMG (2 mg/ml), Col I (2 mg/ml), and Matrigel (5 mg/ml) examined using IF staining of Col I (green) and fluorescence microscopy. Scale bars, 10 μm. **e**–**g** Close-up views of the microstructures of the polymerized TMG, Col I, and Matrigel thinly coated on slides. Scale bars, 1 μm

We have previously reported that the stiffness of porcine breast tissues is about 0.25 kPa [40], in the same range as that of human breast tissues [59, 60] (depending on the measurement methods), and the decellularized breast tissue ECM stiffness is around 0.4 kPa [40], which is similar to the stiffness of Col I gelatinized at the concentration of 2 mg/ml [61]. Based on the native tissue and Col I gel stiffness parameters, we polymerized TMG, Col I, and Matrigel at different concentrations in a 37 °C incubator (5% CO_2_) and measured the stiffness of the gels using atomic force microscopy (AFM). Our results showed that TMG and Matrigel at the concentrations of 2 mg/ml and 5 mg/ml, respectively, polymerized into gels with stiffness similar to that of Col I at the 2 mg/ml concentration (Fig. 1a). The elastic moduli of the gels at these lower concentrations were 0.533 ± 0.291 kPa for TMG, 0.462 ± 0.247 kPa for Col I, and 0.428 ± 0.049 kPa for Matrigel. When the concentrations of TMG, Col I, and Matrigel were increased to 4 mg/ml, 3 mg/ml, and 10 mg/ml, the elastic moduli of the gels reached 1.332 ± 0.189 kPa, 0.787 ± 0.333 kPa, and 1.460 ± 0.247 kPa, respectively. The stiffness of Matrigel polymerized at the 10 mg/ml stock concentration fell into the range of what was previously reported [62], where large lot-to-lot variations were observed. As demonstrated by TMG and Matrigel, doubling the hydrogel concentration roughly tripled the stiffness of the polymerized gels. These data indicate that TMG and Col I gelatinized at a same concentration will result in gels with similar stiffness. In contrast, Matrigel is a slightly softer hydrogel at the concentration of 5 mg/ml compared to TMG and Col I at 2 mg/ml concentration.

With the concentrations of the hydrogels being defined for making culture substrata at native breast tissue stiffness, we set out to investigate the microstructures of the polymerized gels. After TMG (2 mg/ml), Col I (2 mg/ml), and Matrigel (5 mg/ml) were polymerized at 37 °C, the gels were subjected to IF staining using Col I-specific antibodies followed by fluorescence microscopy. The results showed that the collagen fibers in TMG were long and curvy and organized into a complex meshwork with cross-connections on the lattice structures and hardly identifiable ends (Fig. 1b). This phenotype is consistent with that seen in human normal breast tissue, where the collagen fibers were observed to be wavy or curly with heterogeneous structures as defined using second harmonic generation (SHG) imaging [63, 64]. The collagen fibers in Col I hydrogel were straight with identifiable ends and laid out in layers of fringe structures (Fig. 1c). In contrast, the polymerized collagen in Matrigel exhibited circular grid patterns with the hollow areas in the sizes of about 2–10 μm (Fig. 1d). Similar structural features of Matrigel was observed under scanning electron microscopy (SEM) as reported elsewhere [65]. The microstructural differences of the three different hydrogels were more obviously visible on slides thinly coated with the gels (Fig. 1e–g). These results clearly demonstrated the topographical and structural differences of TMG, Col I, and Matrigel at their polymerized states, implicating potential impacts of the physical discrepancies of these hydrogel substrata on the phenotypes of the cells grown on the gels.

### The morphologies of breast cancer cells on the natural hydrogels

It was reported that matrix stiffness, 3D structures, and components are important for the adhesion and morphology of cells grown on the matrix [46, 61]. To define the morphological features of breast cancer cells grown on the physicochemically distinct TMG, Col I, and Matrigel, we polymerized the three types of hydrogels at their respective concentrations that resulted in physiological breast ECM stiffness as described above, and performed IF staining of the cells and collagen I within the matrices. As we observed a phenotype that the cells cultured on the hydrogels tend to invade into the matrices over time (Figure S1), the morphologies of the cells on the matrices were analyzed at 16 and 24 h after cell seeding so that most cells could fully display their morphology and yet were not totally submersed in the gels.

**Fig. 2:**
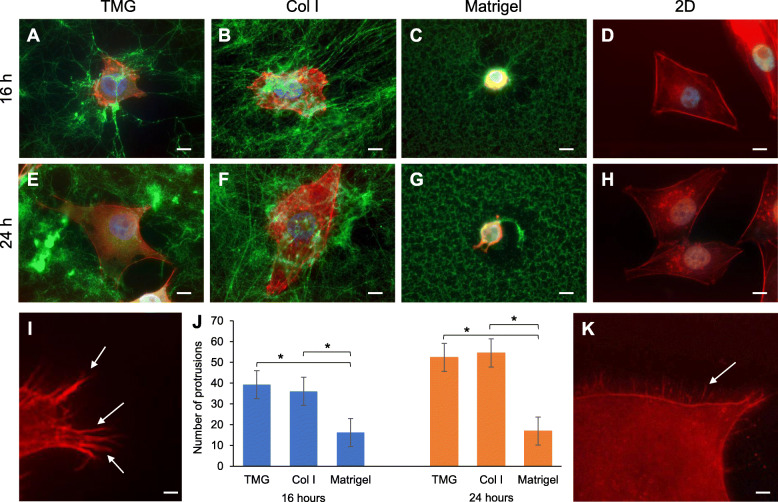
The morphologies of MM231 cells cultured on the three different hydrogels and 2D glass surface. **a**–**d** MM231 cell morphologies on TMG, Col I, Matrigel, and glass slide after 16 h of culture. Col I staining, green; F-actin/phalloidin, red; nucleus/Hoechst, blue. Scale bars, 10 μm. **e**–**h** MM231 cell morphologies on the hydrogels and glass slide after 24 h of culture. Scale bars, 10 μm. **i** Large protrusions from MM231 cells grown on the three types of hydrogels. Scale bar, 1 μm. **j** Quantification of the protrusions from the cells grown on the hydrogels at the 16- and 24-h time points. *P < 0.0001 comparing TMG and Col I to Matrigel at both 16 and 24 h (TMG, 16 h: *t* = 9.912, 24 h: *t* = 9.987; Col I, 16 h: *t* = 8.144, 24 h: *t* = 11.703). There was no significant difference between TMG and Col I at either time. **k** Small, brush-like protrusions; close-up view of a part of the cell shown in **e**. Scale bar, 1 μm

Our results showed that MDA-MB-231 (MM231) breast cancer cells exhibited a spreading morphology on TMG or Col I hydrogel and a round shape on Matrigel (Fig. 2). The round morphology of cells on Matrigel has also been reported elsewhere [66]. Yet, when the cells were grown on slides coated with a very thin layer of Matrigel or cultured for extended hours, they could display shapes that were more spread (data not shown). The morphological differences of the cells on the different matrices were more evident at the time of 24-h (Fig. 2e–g) than at the 16-h time point (Fig. 2a–c). Another distinguishable feature of the cells on the three different matrices is the number and shape of protrusions extended from the cells. More protrusions were seen on the cells grown on TMG and Col I than on Matrigel (Fig. 2a–c, e–g, i) that was a significant phenotype (Fig. 2j). With the stiffness of the gels being set at similar native ECM levels (Fig. 1a), the number and shapes of the protrusions seemed to be correlated to the collagen fiber textures of the matrices. When Matrigel was used at high concentration (~ 8 mg/ml or 10 mg/ml) as a thin layer, the cells could also display more spreading morphology (data not shown). In addition to the major protrusions, some small brush-like protrusions were also visible on the cells cultured on TMG (Fig. 2k). This phenotype was less prominent in the Col I cultures and almost non-visible in the Matrigel cultures, especially at 16 h. By 24 h, there were slightly more brush-like protrusions visible on the cells in the Col I cultures. Though the cells grown on 2D also displayed a spreading morphology (Fig. 2d, h), the bodies of the cells seemed to be relaxed and the cell edges were in more straight and better-defined shapes than those on TMG or Col I (Fig. 2d vs. a and b; h vs. e and f). No clear brush-like protrusions were identifiable on the cells in the 2D cultures (Fig. 2d, h). The same cell spreading phenotypes were also seen in SUM1315 cells grown on the different substrata tested here (Figure S3).

We also noticed a pulling deformation of the hydrogel structures effected by the cells grown on the matrices (Fig. 2a–c, e–g). This phenotype has been noted in other studies [67, 68]. Tumor cells are known to become stiffer by pulling in ECM components, stressing and altering collagen fibers around the tumor [69–71]. So, cell pulling of the hydrogel fibers is logical to see, but was a phenotype missing on the 2D culture surfaces.

### Breast epithelial cells express distinct surface receptors on the different biogels

The ECM of breast tissues is composed of complex ECM proteins, with total collagen accounting for about 80% of the composition and Col I and Col III dominating the total collagen at about 77% as we reported [39, 40] (there is a slight variation of collagen abundance between mouse, porcine, and human breast tissues but within a similar range). Clearly, the non-Col I collagens as well as ECM proteoglycans and glycoproteins comprise a significant amount of breast ECM proteins, implicating potential differences between tissue ECM and collagen I (or other single collagens) on structural and biochemical guidance for cell biological activities. In contrast, Matrigel (lrECM) has very little amount of collagen I and abundant laminin and collagen IV [39, 72]. It is of note, while the normal basement membrane contains more Col IV than laminin [25], Matrigel has a much higher content of laminin than Col IV. The different proteins within ECM serve as ligands for different cell surface receptors whose activations are essential for the induction of various biological activities of the cells.

Based on the compositional and the microstructural differences between TMG, Col I, and Matrigel as well as the distinct morphologies of the cells on the matrices, we speculated that mammary epithelial cells grown on the different hydrogels may express different membrane receptors or express certain receptors at different levels. To test this speculation, an acini formation assay using MCF10A normal breast epithelial cells and an on-gel culture of MM231 breast cancer cells were conducted. The expression of selected cell surface receptors was analyzed using IF staining and microscopy.

**Fig. 3:**
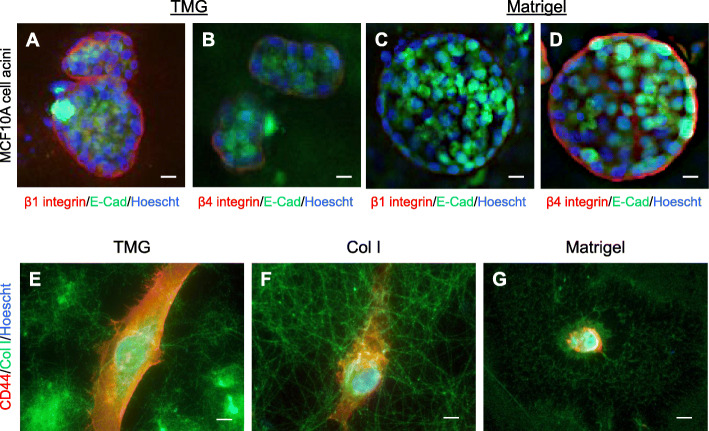
Cell surface biomarker expression. **a**–**d** The MCF10A cell-formed acini in TMG and Matrigel were IF stained with either β1 or β4 integrin (red), E-cadherin (green), and Hoechst (blue). Scale bars, 100 μm. **e**–**g** The expression of CD44 in MM231 cells cultured on the three types of hydrogels were examined using IF staining. CD44, red; Col I, green; nucleus/Hoechst, blue. Scale bars, 10 μm

Normal breast epithelial cells form acini structures on Matrigel, representing a robust in vitro model for mammary gland mimicry, that is hardly seen in cancer cell cultures where the cells form irregularly shaped clusters [73, 74]. Acini formation on Matrigel can either present as a dense cluster or a ring structure formed by cells grown on the gel, depending on the status of cell death in the center of the acini [75–78]. As TMG contains limited amounts of laminin and Col IV, the acini formed within TMG is overall much less (data not shown) and at smaller sizes compared to those in Matrigel (Fig. 3a–d). Consistent with high Col I/Col III and low laminin levels in TMG, the MCF10A cell membrane β1 integrin receptors that mainly use Col I or/and Col III as binding ligands in the matrix were expressed at high levels whereas the β4 integrin receptors that prefers laminin as matrix ligands were detected at very low levels (Fig. 3a, b, red color). This contrasting phenotype is clearly displayed via the surfaces of the cells in the outer layer of the acini structures that were in contact with the surrounding hydrogel. Oppositely, β1 integrin receptors were barely detectable while β4 integrin receptors were highly expressed on the acini formed in Matrigel (Fig. 3c, d). The cell surface E-cadherin (E-cad), known for mediating cell-cell adhesions, was similarly expressed on the interacting cells within the acini formed on the two types of hydrogels (Fig. 3a–d, green color).

To demonstrate cancer cell membrane marker protein expression on the different hydrogels, MM231 cells were seeded on polymerized TMG, Col I, or Matrigel as before; cultured for 24 h; and IF stained for the cell surface adhesion molecule CD44 (also called homing-associated cell adhesion molecule, or HCAM). CD44 is a receptor involved in cell-cell and cell-matrix interactions [79]. It is highly expressed in many human cancers and plays an important role in breast cancer metastasis [80–82]. Its matrix ligands include collagen, fibronectin, laminin, hyaluronic acid, osteopontin, and metalloproteinases. Our results showed that CD44 expression was higher in MM231 cells cultured on TMG and Col I hydrogels than on Matrigel (Fig. 3e–g). This phenotype was more obvious in the samples cultured longer than 24 h when the cells on the matrices, especially those on Matrigel, spread better (Figure S2). The same CD44 staining patterns were also seen in SUM1315 cancer cells grown on the hydrogels (Figure S4). These data collectively indicate that mammary epithelial cells grown on TMG, Col I, and Matrigel express quite distinct membrane receptors even when the gels were at similar physical stiffness.

### Cancer cell differentiation profiles on the hydrogels

The differentiation status of the cancer cells within breast tumors is a common indicator for breast cancer grading and is closely linked to cancer aggressiveness, prognosis, and therapeutic planning. The grade of cancer cell differentiation reflects the degree of resemblance of the malignant cells to normal breast epithelial cells [83]). High-grade cancer cells are morphologically distinct from normal cells and are generally associated with more aggressive cancer types compared to well-differentiated epithelial cells. To better define cancer cell differentiation status, a set of molecular markers such as Vimentin (Vim), E-cadherin (E-cad), and Zonula Occludens-*1* (*ZO-1*, also called *Tight Junction Protein 1 or TJP1*) are commonly used in immunostaining of cancer cells [84–86].

**Fig. 4:**
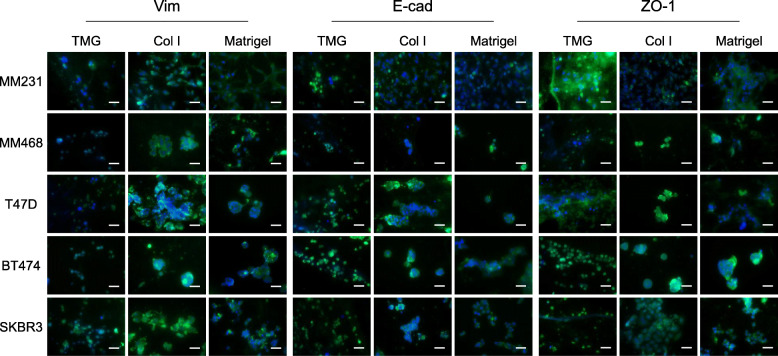
Differentiation profiles of the different subtypes of breast cancer cells on the hydrogels. Immunofluorescence antibodies were used to detect the expression of the differentiation markers E-cad, Vim, and ZO-1 (green) in the cancer cells cultured on TMG, Col I, and Matrigel. The nuclei of the cells were stained blue with Hoescht dye. Fluorescence microscope objective × 40. Scale bars, 50 μm

Since MM231 cells displayed different morphologies and plasma membrane receptor expression on TMG, Col I, and Matrigel (Figs. 2 and 3), we speculated on the possibility of breast cancer cells exhibiting divergent differentiation status on the physicochemically distinct matrices. To interrogate this assumption, five breast cancer cell lines, MM231, MM468, T47D, BT474, and SKBR3 representing the basal B/claudin-low, basal A, luminal A, luminal B, and HER2-overexpressing molecular types of breast cancers, respectively, were cultured on the three types of hydrogels for 5–7 days and stained with Vim, E-cad, and ZO-1. The results showed that Vim expression is overall lower in all the types of cancer cells cultured on TMG than those on Col I or Matrigel and is comparable between cells cultured on Col I and those on Matrigel (Fig. 4, left panels). In contrast, E-cad and ZO-1 expression at the sites of cell-cell contacts are highest in the different types of cancer cells cultured on TMG, a bit lower in the Col I culture groups, and lowest in the Matrigel cultures (Fig. 4, middle and right panels) although the cancer cells tended to be clustered together in the Col I and Matrigel cultures. The expression of E-cad and ZO-1 on the membranes of the MM231 cells cultured on Matrigel was almost non-detectable. Our data on the expression of the differentiation markers in MM231 and SKBR3 cells are mostly in agreement with a previous observation elsewhere [87].

Another interesting phenotype we observed during the cell differentiation experiments was that the cancer cells grown on TMG generally appeared smaller, without much cell clustering (Fig. 4, the left column of each marker panel), which is apparent in the MM468, T47D, BT474, and SKBR3 groups and less so in the MM231 group (Fig. 4, the middle and right columns of each marker panel). The cancer cell clustering phenotype was consistent with a previous observation where certain cancer cell lines or primary cancer cells formed spherical shapes and were depleted of normal differentiation phenotype in Matrigel cultures [88]. Additionally, the shapes and sizes of the cancer cells grown on Matrigel seemed to be more diverse than those of the cells on TMG or Col I (Fig. 4, the right column of each marker panel). These data collectively indicate that the three types of hydrogel matrices direct breast cancer cells to distinct levels of differentiation states as exhibited by the expression or loss of the cellular differentiation markers.

### Cancer cell migration and invasion in the hydrogel cultures

Spatial migration and invasion are closely linked characteristics of invasive cancer cells living in native tissues, where the stiffness, composition, and topography of the tissue ECM are all mediators of the processes [29, 89, 90]. These natural properties of the native microenvironment are barely preserved in 2D plastic or glass culture vessels, and cell migration is limited to the flat surface of culture without concomitant invasion. As we have observed, cancer cells grown on hydrogel not only have a gripping effect on the surrounding matrix but also migrate and invade into the gel (Figure S1). To further examine and decouple the migration and invasion capacities of cancer cells on physicochemically different hydrogels, we have developed two assay systems for quantitative analyses of the spatial motility of the cells.

**Fig. 5:**
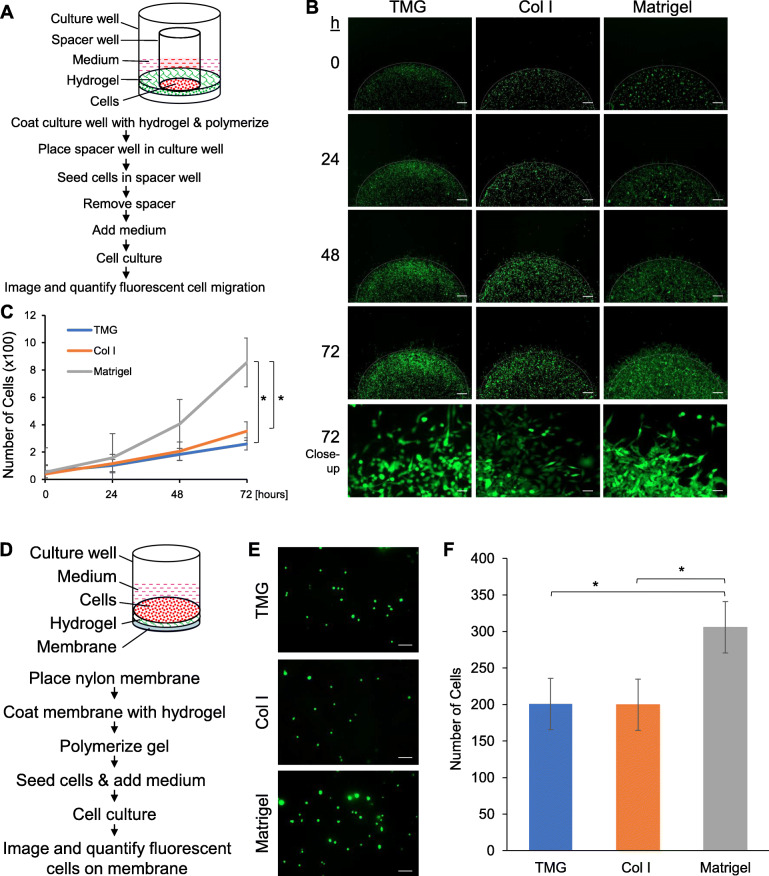
Breast cancer cell migration and invasion on the hydrogels. **a** Diagram of the experimental setup for the migration assay. **b** GFP-MM231 cell migration on top of the matrices over 72 h. Scale bars, 500 μm. The bottom panel shows representative migration line at × 10 magnification. Scale bars, 100 μm. **c** Quantification of cell migration outside the defined circle region on each matrix over time. *P ≤ 0.001 comparing TMG and Col I to Matrigel after 72 h (TMG, *t* = 5.586; Col I, *t* = 4.907). There was no significant difference between TMG and Col I. **d** Diagram of the experimental setup for the invasion assay. **e** Representative views of GFP-MM231 cells under a fluorescent microscope after invasion through matrices onto Nytran membrane (× 10 magnification). Scale bars, 100 μm. **f** Quantification of cell invasion onto membrane. *P < 0.05 for TMG and Col I compared to Matrigel after 24 h (TMG, *t* = 3.200; Col I, *t* = 2.273). There was no significant difference between TMG and Col I. Error bars in **c** and **f** represent mean ± SD of three independent experiments

In the cell migration assay, cell culture wells were coated with TMG, Col I, or Matrigel followed by placing a spacer well in the middle of the culture well after gel polymerization and cell seeding in the spacer wells (Fig. 5a). Cell migration on the matrices toward outside of the seeding areas was observed after the removal of the spacer wells. The results showed that MM231 cells progressively migrated on the matrices, with a visible increase in occupancies outside their initial seeding regions over time (Fig. 5b). Interestingly, while the cells migrating on TMG or Col I were mostly as individuals, the ones on Matrigel moved more in groups, clusters, lines, or a rallied migrating front, although a couple of individual migrating cells were also seen sporadically in the migration direction (Fig. 5b, bottom panel), displaying an overall pattern resembling collective cell migration as described elsewhere [91]. Quantitative analysis of the number of cells migrated into the surrounding regions revealed similar migration rates of the cells cultured on TMG and Col I and a much greater abundance of migrated cells on Matrigel (Fig. 5c). These data indicate that cancer cells have quite different migration capacities and may utilize different migrating strategies on structurally and biochemically different matrices.

To assess cancer cell invasion on the different hydrogels, a Nytran N membrane was placed at the bottom of a culture well and coated with 50 μl of TMG, Col I, or Matrigel, followed by seeding GFP-MM231 cells on top of the polymerized gel (Fig. 5d). This thick layer of gel provides an adequate barrier for the cells to penetrate through; a culturing time of only 24 h makes it less likely for cell proliferation to have an impact on the data, as others have observed [92]. The invasion of the fluorescent cells through the gel and blotting onto the membrane was examined, after removal of the gel, under a fluorescent microscope and quantified using ImageJ software. Our results showed that the cancer cells were able to penetrate all the three types of hydrogels to some extent after 24 h. Consistent with the migration assay data, a similar number of cells invaded the TMG and Col I hydrogels while a greater number of cells penetrated through the Matrigel onto the membrane (Fig. 5e, f). These data indicate that the cancer cells move faster in the laminin-rich Matrigel than in the fibrillar Col I-rich gels.

### Lactate is a major metabolite of breast cancer cells grown on hydrogel and controls cell proliferation

As a key energy source for cellular metabolism, glucose is essential for cell survival and growth even under reprogrammed cancerous conditions [93, 94]. Most normal cell lines and cancer cells have adapted to utilize glycolytic metabolism of glucose to lactate as a quick energy-obtaining route both in anaerobic and aerobic environments (Warburg effect) [95, 96]. Additionally, it has been shown that glucose concentration is a more important factor than serum concentration in culture medium for cell survival and growth [97]. Utilizing these glucose metabolic characteristics, we have examined the presence and extent of carbon incorporation from glucose into metabolites of HUMEC or MM231 cells grown on TMG, Col I, and Matrigel in the carbon-13 (^13^C)-labeled glucose-containing and serum-free culture medium using nuclear magnetic resonance (NMR) spectroscopy. NMR is able to distinguish natural carbon-12 (^12^C) incorporation from ^13^C incorporation into metabolites. For biological relevance, a glucose concentration of 1 g/l at human physiological levels was used in the experiments. The spectra for the medium-only samples without cell cultures were used as background reference controls (Figure S5), and the spectra for the samples of the hydrogel-coated with or without HUMEC or MM231 cell cultures for 1 h after cell seeding served as baseline culture controls (Figure S6), which revealed very little difference among and between the samples. No evidence of active metabolism could be found in any of the controls. There were slight differences in chemical shifts in a few of the control spectra, presumably due to pH or polarity differences in the media or hydrogels of controls containing different cell types and/or scaffold types (Figure S6). The spectra data of the 7-day cultures were compared for differences of major metabolic changes among and between the experimental groups (Figure S7).

**Fig. 6:**
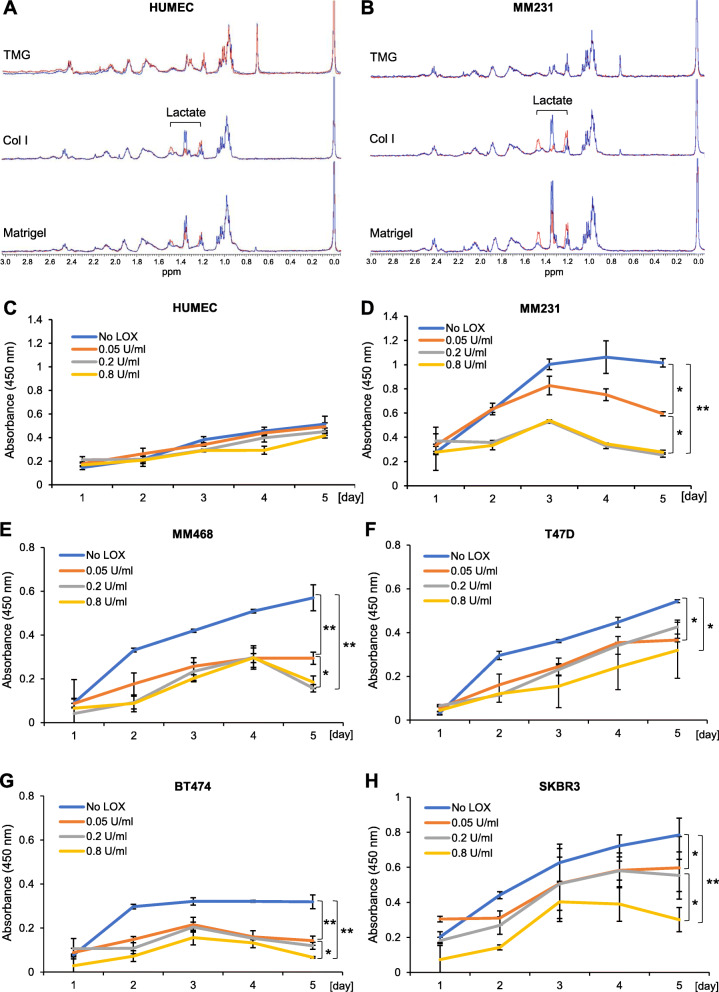
Mammary epithelial cell metabolic changes and response to lactate depletion on the hydrogels. **a**, **b** NMR spectroscopy data of major metabolite changes in the culture media of HUMEC or MM231 cells grown on the three types of hydrogels for 7 days. HRMAS spectra, blue = decoupled proton spectra; red = ^13^C-^1^H coupled spectra. **c**–**h** Effect of lactate depletion from the culture medium on the proliferation of HUMEC or the different subtypes of breast cancer cells grown on Matrigel. Error bars represent mean ± SD of three independent experiments, with each experiment having triplicate samples. There were no significant differences in the proliferation of the HUMEC cells cultured on the different gels. MM231 cells: *P < 0.05 for 0.05 U/ml versus 0.2 or 0.8 U/ml (*t* = 18.172 and *t* = 19.492) and 0.05 U/ml versus no LOX (*t* = 15.280), respectively, and **P < 0.001 for 0.2 U/ml and 0.8 U/ml versus no LOX (*t* = 30.454 and *t* = 26.801). There was no significant difference between the 0.2 U/ml and 0.8 U/ml groups; MM468 cells: *P < 0.05 for 0.05 U/ml versus 0.2 and 0.8 U/ml (*t* = 7.268 and *t* = 4.577), **P < 0.001 for no LOX versus 0.05, 0.2, and 0.8 U/ml (*t* = 6.865, *t* = 8.387, and *t* = 9.639); T47D cells: *P < 0.05 for no LOX versus 0.05, 0.2, and 0.8 U/ml (*t* = 26.869, *t* = 6.242, and *t* = 3.380). There was no significant difference between the 0.05, 0.2, and 0.8 U/ml groups; BT474 cells: *P < 0.05 for 0.8 U/ml versus 0.05 and 0.2 U/ml (*t* = 6.038 and *t* = 5.201), **P < 0.001 for 0.05, 0.2, and 0.8 U/ml versus no LOX (*t* = 7.388, *t* = 9.115, and *t* = 12.747). There was no significant difference between the 0.05 U/ml and 0.2 U/ml groups; SKBR3 cells: *P < 0.05 or **P < 0.001, comparing all concentrations of LOX except for between 0.05 U/ml and 0.2 U/ml, which had no significant difference (no LOX vs. 0.05, 0.2, and 0.8 U/ml, *t* = 3.807, *t* = 2.817, and *t* = 7.523, respectively; 0.8 U/ml vs. 0.05 and 0.2 U/ml, *t* = 2.655 and *t* = 3.7587)

In the culture medium of HUMEC or MM231 cells grown on TMG, no lactate accumulation was detectable (Fig. 6a, b, upper panels). An increase in intensity of the doublet at 1.34 ppm, corresponding to lactate accumulation, was observed as the major change in the spectra of both Col I and Matrigel samples (Fig. 6a, b, middle and bottom panels) relative to the control spectra (Figures S5 and S6), with the Matrigel cultures exhibiting slightly higher lactate levels compared to those of Col I. Overall, MM231 cells cultured on Col I and Matrigel had more lactate released into the culture medium compared to HUMEC grown on the same matrices (Fig. 6a, b, middle and bottom panels). These data are consistent with the proliferation profiles of mammary epithelial cells grown on the three types of hydrogels we previously reported [40], where the cells proliferated the most on Matrigel, intermediate on Col I, and the least on TMG. In the decoupled spectra (blue peaks), ^13^C-labeled lactate was not distinguished from natural abundance ^12^C. However, in the corresponding ^13^C-coupled spectra (red peaks, decoupler off), new peaks for metabolites having incorporated ^13^C were clearly seen for the incorporation of ^13^C-labeled carbon into lactate, by observing the ^13^C satellites for lactate at 1.47 ppm and 1.21 ppm, respectively (Fig. 6a, b, middle and bottom panels).

Moreover, the small red center peaks at 1.34 ppm in the coupled spectra of the Col I samples for both HUMEC and MM231 cells revealed lactate having trace abundance of natural ^12^C (Fig. 6a, b, middle panels). In the Matrigel groups, a great deal of natural ^12^C incorporated into the lactate metabolite (Fig. 6a, b, bottom panels). Examination of the Col I or Matrigel plus medium and the 1-h cell culture on Col I or Matrigel controls revealed no lactate presence prior to the extended 7-day culturing of the HUMEC or MM231 cells (Figure S6 and Fig. 6). Thus, the residual ^12^C-lactate had come from a non-^13^C-enriched source of carbon during the incubation period. It could exist in the Col I or Matrigel matrix prior to tissue culture or carried by the cells into the cultures. Nevertheless, the largest pool of carbon incorporated into lactate, in the HUMEC and MM231 cultures on Col I and Matrigel, comes from ^13^C-labeled glucose. In the result for HUMEC on Matrigel cultures (Fig. 6a, bottom panel), the overlaid decoupled (blue) and coupled (red) spectra show similar accumulation of lactate, both labeled and unlabeled. Yet, both ^12^C- and ^13^C-lactate were observed in roughly equal amounts after 7 days of culture. This data implies that ^12^C-glucose was present in Matrigel as received. However, NMR signals of free glucose are at or below the level of detection in all of the samples, making it difficult to determine the presence of natural abundance ^12^C-glucose in the Matrigel controls.

The NMR spectrum of MM231 cells grown on Col I (Fig. 6b, middle panel) showed that the lactate formed during the culturing period contained predominantly ^13^C-labeled carbon and is therefore due mostly to the metabolism of ^13^C-labeled glucose from the medium. In contrast, the MM231 metabolites in the Matrigel cultures (Fig. 6b, bottom panel), as shown by the overlay of the ^13^C-coupled spectrum (red) and fully decoupled spectrum (blue), revealed that roughly half of the lactate formation was due to natural ^12^C-incorporation into the lactate pool. In fact, integration of the two spectra (coupled vs. decoupled) confirms that the resulting ^13^C-lactate is 45% of the total carbon incorporated into lactate during incubation, within experimental error. Overlaying the two spectra therefore gave a clear and convenient representation of the contributions of both ^12^C and ^13^C into the lactate metabolite pool during incubation. When the same analysis was applied to the 7-day HUMEC on Matrigel culture results (Fig. 6a, bottom panel), ^13^C was found to be only 28% of the total carbon incorporated into lactate during the incubation period. Clearly, in contrast with HUMEC cells, MM231 cells incorporated more ^13^C from glucose for metabolic activities when cultured on Matrigel. Matrigel appears, by simple proton NMR, to provide either or both a metabolic carbon source and/or unidentified growth factors.

Accumulating evidence has indicated that glycolysis-generated lactate, which was ever thought a waste product of the glucose anaerobic metabolism, can actually be used as an energy source by cancer and other cells for the production of pyruvate that is in turn utilized in the TCA cycle [98]. We speculated that the cells grown on Col I or Matrigel matrices may utilize the lactate they generated as a major nutrient to support their survival and growth. To test this hypothesis, we depleted lactate in the culture medium of HUMEC or MM231 cells grown on Matrigel, where the cells grew faster than on TMG or Col I [40], as a proof of principle, using lactate oxidase (LOX) and measured proliferation of the cells. Our results showed that LOX dose-dependently attenuated the proliferation of both HUMEC and MM231 cells (Fig. 6c, d). Interestingly, LOX treatment decreased MM231 proliferation to a much larger extent compared to HUMEC proliferation, which seemed to be less sensitive to increasing doses of LOX than MM231 cells. No significant differences in proliferation were observed between the LOX-treated and non-treated HUMEC cells grown on Matrigel. Higher dose of LOX totally abolished the proliferation of HUMEC and MM231 cells grown on Matrigel in 5 days. To examine the suppressing effect of LOX on the propagation of breast cancer cells other than the basal B type MM231 cells, we further performed cell proliferation assays using MM468, T47D, BT474, and SKBR3 cells of the basal A, luminal A, luminal B, and HER2-overexpressing breast cancer types on Matrigel. The results showed that, although there were variations of the cell growth rates and the degrees of cell sensitivities to LOX across the different types of cancer cells, the proliferation of the cells was inhibited by LOX at the same concentrations tested in MM231 cells (Fig. 6e–h). High concentration of LOX almost totally abolished the proliferation of the cancer cells tested except the T47D cells, which seemed to be slightly less sensitive to LOX treatment compared to the other types of the breast cancer cells. These data demonstrated that LOX is a broad inhibitor of breast cancer cell proliferation and suggest an essential role of glycolytic lactate production in promoting proliferation of the cells, especially cancer cells, in the hydrogel cultures.

## Discussion

This study systemically examined the biological aspects of three types of native ECM hydrogels: TMG, Col I, and Matrigel. While Col I and Matrigel have been widely used in the biomedical field for various 2D or 3D cultures, normal native full ECM hydrogel derived from animal or human tissues [39, 40, 99], such as TMG, still requires better characterization for tissue- and disease-specific research applications. One of the mechanical properties of hydrogel important for tissue mimicry and cell biology studies is stiffness, as different tissues have quite different stiffness. For instance, the stiffness of normal breast tissue expressed in Young’s modulus is about 0.1–4 kPa and that of skin, fascia, muscle, and lateral ligaments around breast tissues are 1–20 kPa, 80–250 kPa, 10 kPa, and 100 kPa, respectively [59], and different measurement and analytical methods may give rise to variable values [100, 101]. Breast tumors, on the other hand, have stiffness at 10–42 kPa [102], about 3–13-fold harder than normal breast tissues and 7 × 10^4^–3 × 10^5^ times softer than that of 2D polystyrene plastic surface [61]. Since the stiffness of a culturing substratum can significantly affect cellular activities [47], caution needs to be taken when applying hydrogel systems to investigate biological phenotypes and mechanisms related to a disease of a specific tissue.

To observe mammary epithelial cell phenotypes on TMG, Col I, and Matrigel at physiological stiffness, we defined the concentrations of the hydrogels that allowed the elastic moduli of the polymerized gels to be comparable to the decellularized breast tissue ECM as we reported before [40]. This procedure avoided the potential influence of stiffness disparities of the different gels on cell phenotypes and provided reference parameters for future studies addressing modifications of tissue ECM by cultured cells. Under the similar stiffness conditions, the microscopic structures of the collagenous fibers of the TMG, Col I, and Matrigel matrices were strikingly distinct from one another. Overall, there was greater similarity between Col I and TMG fibrillar structures than with Matrigel, and TMG had complex interlinked fibrous networks compared to Col I-alone fibers. These phenomena are very likely due to the innate nature of the biochemical properties of the gels. In other words, the different ECM proteins within the gels form interlinked spatial structures based on their polymerizability and native roles as structural or non-structural proteins. For instance, the normal breast ECM-derived TMG contains not only abundant Col I but also a good amount of Col III, Col V, and Col VI in addition to other glycoproteins and proteoglycans [39, 40].

Col III is the second most abundant collagen in breast tissues [39, 40] and regulates the fibrillogenesis and diameter of Col I fibrils [103]. It can heterotypically copolymerize with Col I within the same collagen fibril via intermolecular crosslinking to affect the tensile properties of tissues [104, 105] and may form fine reticular fibers in association with Col V, other glycoproteins, and proteoglycans [106]. Importantly, the ratio of Col I to Col III within a tissue is closely related to the rigidity of the tissue and can change in many tissue-related pathologic conditions [107]. It was reported that Col III played a suppressing role in tumor development, cancer cell proliferation, and metastasis, presumably through regulating the density and alignment of fibrillar collagen [108]. With the interactions of the fibrillar collagens and other ECM components, it is not unexpected that the frameworks of TMG collagen fibers were more intricate than the microstructures of the Col I-only and collagen-low Matrigel as demonstrated in Fig. 1. The visually straight and long collagen fibers in the polymerized collagen I gel had various longitudinal, transverse, or horizontal crossings as seen in tendon fibrils [109], with a lack of inter-fiber connections, making the architectures of the collagen fibers different from those of TMG. The polymerized Matrigel, however, exhibited a quite distinct nonfibrillar lattice structure, with patches of Col I unevenly distributed in the walls of the networks. This is probably due to the abundant laminin, which is a four-arm glycoprotein [110], co-polymerized with other main components such as Col IV and nidogen/entactin in Matrigel to cast the frame of the lattice. In this framework, maybe Col I plays an assisting role in reinforcing the architecture because of its minor abundance in Matrigel. It seems both the lack of fibrillar collagens and the rich nonfibrillar laminin in Matrigel contribute to the hive-like structures upon the polymerization of the gel. Matrigel is a good tool for basement membrane mimicry and studies needing laminin-, Col IV-, or nidogen-rich environments. Yet, its underdefined mouse sarcoma cell-derived constituents and growth factor contents (e.g., NGF, EGF, TGFβ, and VEGF ranging from about 5- to 175-folds more than that of serum levels depending on growth factor depletion conditions) may have some impacts on cell signaling and disease-specific studies.

The topographical and microstructural differences of the polymerized TMG, Col I, and Matrigel implicate the distinct mechanical forces that these matrices may apply to the cells grown on top. Conversely, the tangibility and the deformability of the gels, different from the hard plastic or glass substratum, allow the cells to employ their natural adhering, spreading, mobilizing, molecule-secreting strategies to flexibly interact with and modify the structures of the matrices for best dwelling, survival, growth, and movement. These matrix-cell mutual interactions guided by the topography and structures of the matrices as well as the biochemically dissimilar ECM proteins, which serve as selective gripping ligands or signaling initiators for the cells, in TMG, Col I, and Matrigel clearly contribute to the distinguishable morphological features of the cells on the matrices (Fig. 2). Supporting our data, cell adhesion, morphology, and proliferation differences on Col I, laminin, and fibronectin were observed elsewhere to be matrix ligand-specific [111].

The larger protrusions from the cell membrane are known as pseudopodia and the brush-like ones extending out from lamellipodia as filopodia or microspikes. The formation of these protrusions is associated with cellular sensing mechanisms and involves different cell adhesion and cytoskeleton organization strategies [112, 113]. These protrusions, connected with cytoskeleton proteins, are essential for the cells to sense the environmental footing points and generate pulling forces for migration once focal adhesions are established. The pulling effect shown on the gel matrices induced by the contraction of the cells and the footing attachments of the cell protrusions on the matrix fibers are clear indications of the cell-matrix interactions. These visible signs of mutual interactions are invisible in the 2D cultures, where the adaptive plasticity of the cells dominates the interactions. The irregular shapes and the protrusions of the cells grown on the TMG and Col I hydrogels seem to reflect the weaving complexity of the fibrillar structures of the gels compared to a 2D surface, which favors the cells to display a more relaxed and spreading morphology without much of the spiky protrusions under the same culturing conditions. The sparse protrusions of the cells on Matrigel were coincident with the nonfibrillar and hive-like structures of the gel matrix, suggesting that the matrix fibers are responsible for the induction of the protrusions. This notion is supported by an in vivo observation that collagen fibrils seem to serve as scaffolds for cancer cells to align themselves, maintain their anchorage, and acquire optimal shape [114]. Given a hydrogel environment created for most of cell cultures is considerably even in stiffness, other mechanical properties, and structures throughout the gel, one would expect to see more variations of cancer cell morphologies in a pathologically heterogeneous tumor tissue. The various cell protrusions and matrix alterations illustrated in the hydrogel cultures mirror a similar picture of how the cells act in native tissues.

Within a tissue microenvironment, the resident cells are in close contact with tissue ECM in all directions. Not only the mere physical support but also the topological, architectural, and compositional properties of the ECM affect the presence of cell surface receptors for binding to ECM ligands. This context-dependent cell-ECM spatial interaction can be well recapitulated in acini formation assays as shown in Fig. 3. It is interesting that while a laminin- and Col IV-rich environment has been considered essential for maintaining acini structures [115, 116], the laminin- and Col IV-low TMG also supported acini formation but to a much less degree compared to Matrigel. This implicates that there are ECM factors other than laminin and Col IV potentially involved in promoting the formation of acini structures or that the small amount of laminin, Col IV, and other glycoproteins and proteoglycans in TMG was able to maintain a minimal requirement for acini formation. Future works on testing acini formation in TMG by supplementing certain basement membrane components or growth factors may provide further insights into acini development and maintenance in native tissue environments.

As critical focal adhesion molecules, integrins in the form of transmembrane heterodimers selectively connect ECM ligands with cytoskeletal structures and intracellular focal adhesion regulatory proteins via their beta (β) subunits to conduct outside-in and inside-out signals for matrix remodeling, degradation, stiffness regulation, cytoskeleton organization, and many cellular activities. The β1 integrin subunit is a major cell surface receptor for collagens and may also recognize laminins and fibronectin as ligands [80]. A strong expression of β1 integrin at the interface of the polarized MCF10A cells and TMG and a very low expression in the Matrigel acini in our data (Fig. 3a–d) seem to indicate that β1 integrin prefers the major ECM collagens as ligands. This is in clear contrast with the β4 integrin subunit, which exhibited strong affinity toward the laminin- and Col IV-rich Matrigel. As integrins play important roles in cancer cell survival, proliferation, migration, and response to anti-cancer drugs [117], the species and expression levels of cell membrane integrins in the different matrices are therefore critical for understanding cancer cell biology in specific microenvironments and designing therapeutic drugs that have selective targeting efficacies.

Similar to integrins, CD44 is a transmembrane glycoprotein that serves as a receptor for hyaluronic acid, osteopontin, collagens, fibronectin, and laminin. By interacting with the ECM proteins, CD44 facilitates the maintenance of the structures of both ECM and cells. Variations of the expression of CD44 isoforms have been implicated in tumor development [79]. The abundant expression of CD44 in the cells cultured on TMG and Col I but not those on Matrigel (Fig. 3e–g), even when the cells were better spread over extended culturing time (Figure S2), tends to indicate that the receptor prefers the fibril-rich or collagenous matrix rather than the nonfibrillar laminin or Col IV substratum for better display to facilitate cell-ECM interactions. This concept is supported by the observations that CD44 was found to be in complex with integrins and other integrin-interacting cell surface receptors such as HER2, NG2, and CXCR4 [118–120]. Thus, our data may provide reference for future studies addressing differential expression of CD44 isoforms and isoform-specific cross talks with other receptors in normal or cancerous cells grown on compositionally, structurally, and mechanically different ECM.

The living microenvironment of mammary epithelial cells is important for the differentiation and the maintenance of the differentiation states of the cells both in native tissues [121] and in cultures [122]. As key physical components of natural tissue microenvironment, ECM and its constituent collagen were suggested to be required for mammary epithelial cell differentiation [123–125]. Our data of the differentiation marker expression in the breast cancer cells grown on the three different types of hydrogels (Fig. 4) further suggest that the microstructurally and biochemically distinct ECM also mediate cancer cell differentiation, with yet quite distinguishable levels of the molecular marker expression and appearances of cell morphology, especially between the TMG and the Matrigel culture groups. Of the three differentiation markers tested in this study, Vim is a cellular cytoskeleton intermediate filament protein actively expressed in mesenchymal cells or those undergoing cancerous transformation. Its expression is associated with poor differentiation states of epithelial cells [126], commonly seen in many cancer cell lines and cells of invasive tumors, and can be used as a diagnostic indicator of patient’s survival [127–129]. Interestingly, all the five breast cancer cell lines grown on TMG had diminished Vim expression whereas those on Col I and Matrigel expressed higher levels of Vim (Fig. 4), suggesting a differentiation-promoting function of TMG and a transition of the cancer cells from mesenchymal to epithelial states in the TMG culturing environment.

E-cad and ZO-1 are cell membrane-associated proteins involved in direct interactions between neighboring cells and the maintenance of membrane apico-basal polarity, with the former an adherens junction molecule essential for cell-cell adhesion and the latter a tight junction molecule regulating the function of the junction barrier via claudins and occludin and also mediating the assembly of adhesens junction [67, 130, 131]. Interestingly, the expression of E-cad and ZO-1 in breast cancer cells seem to be correlated, and reduction of their accumulation at cell-cell junctions appears to be linked with poor cell differentiation, cancer progression, and metastasis [84, 132]. In our matrix regulation of breast cancer cell differentiation experiment, we observed high, moderate, and low E-cad and ZO-1 expression on the membranes and at the sites of cell-cell contacts of the cancer cells cultured on TMG, Col I, and Matrigel, respectively (Fig. 4). These expression patterns seem to match well with the nature of the three types of matrices. It is possible that the normal breast tissue-derived TMG is capable of restoring the differentiation function of the cancer cells and reformat the ways they interact in the spatial context. On the other hand, the pure Col I matrix may not be able to provide the extra microstructural and biochemical ligand binding support as TMG has for the cancer cells to fully display their markers and differentiation status. Compared to TMG and Col I, Matrigel naturally carries certain ligands and growth factors native to mouse sarcoma tissues but has minimum collagen and other major ECM protein contents [37, 39], and therefore may maintain the dedifferentiation states of the cancer cells better than TMG and Col I, as reflected by the expression levels of Vim, E-cad, and ZO-1 in the cancer cells.

In addition, the differentiation status of the cancer cells on the different matrices is consistent with the proliferation [40], migration, and invasion (Fig. 5) capacities of cells as exemplified by the MM231 cell cultures. Since cancer cells counteractively modify its living microenvironment, it is plausible that the cancer cells cultured in TMG or Col I might gradually develop toward the differentiation, proliferation, migration, and invasion status as seen in Matrigel over extended culturing times or that the normal matrix environment could halt the cancer cells temporarily at a less aggressive state or even convert some of them to “normal” epithelial cells. Future works in this direction will add new insights into our understanding about ECM microenvironment regulation of cancer progression.

The different microstructures of TMG, Col I, and Matrigel (Fig. 1), cell morphology, and protrusion styles (Fig. 2) as well as expression of cell surface adhesion receptors (Fig. 3) were all indicators supportive to the different migratory phenotypes and capacities of the cancer cells on the hydrogels. The fibers formed in polymerized TMG or Col I likely provide better footholds for the cells, compared to those on Matrigel, to cling to through the clusters of membrane integrins or other receptors such as CD44. The fibrillar collagen structures may facilitate cell attachment, pulling, and propelling toward the direction of their movement. This notion is consistent with the observation that cancer cells can realign collagen fibers whose radial structures were designated as tumor-associated collagen signatures (TACS) and could facilitate cancer cell migration and invasion along the fibers [133, 134]. Our migration assays showed that cancer cells grown on TMG and Col I seemed to migrate in the direction along the axes of the spindle-shaped cells and the directionality of the axes were in line with the longer cell protrusions (Figs. 2 and 5), which are important for the 3D migration of the cells [112]. Without a chemoattractant source as existing in native tumors [135], the migration of the cancer cells on the fibrillar TMG and Col I matrices could be random depending on the trajectories of the polymerized collagen fibers. Future works on cancer cell chemotactic migration on the fibrillar matrices may further differentiate the microstructural guidance employed by TMG and Col I to the migrating cells. On the other hand, round-shaped or non-spindle-shaped cells were also seen among the leading-front migrating cells (Fig. 5b, bottom panel). These seemingly non-migrating cells could either be in different stages of migration or adopt different migrating strategies since they may alter their shapes according to the spatial structures of the matrix they were in [136].

Interestingly, the migration and invasion of the cells on TMG and Col I was slower and less penetrable than those on Matrigel. This could be due to the highly expressed integrin receptors on the surface of the cells impeding cell body translocation as observed by Doyle and colleagues [29]. Additionally, the intricate structures of TMG and Col I fibers may be physical barriers for the migrating cells, which may need to modify the fibers to create better alignment for their migration or apply some other strategies for further movement. Furthermore, cancer cells can produce proteolytic enzymes to degrade their surrounding matrix for migration and invasion [90]. It is plausible that cancer cells on the less fibrillar Matrigel require less effort to move through the matrix owing to less abundant receptor gripping on ECM structural ligands, weaker fibrillar barriers, easier degradation of the ECM, and better stimulation by growth factors or other tumor constituents intrinsic to the matrix. All of these physicochemical factors can change the density and mechanics of the matrix, causing alterations in a battery of microstructural parameters of the matrix such as ligand density, pore size, fiber thickness and alignment, or even local stiffness to facilitate cell migration in an anisotropic fashion with dissimilar velocity and self-correlation processes in different directions [137]. The local adjustment of ECM microstructures managed by the cancer cells may form sequential gradients of stiffness and mechanical forces for the cells to undergo durotaxis, a process that favors the cells migrating toward stiffer contacts [138]. This may also explain the stronger trend of cancer cell invasion in Matrigel compared to that in TMG and Col I toward the hard bottom surface of the culturing vessel as demonstrated by the invasion data (Fig. 5d–f). As Matrigel is a matrix deficient in fibrillar structures and contains certain growth factors and other underdefined factors that may facilitate cell migration and invasion, future works measuring cancer cell motility within ECM hydrogel derived from breast cancer tissues along with that from normal breast tissues will not only reveal the microstructural and compositional differences between the pathological and physiological ECM gels but also add tissue-specific insights into whether cancerous ECM facilitates cancer cell spatial motility, and potentially, invasion and metastasis within native tissues.

Although breast epithelial cells spread, migrate, and invade well on TMG, it seems that the glucose-associated metabolic activity of the cells is very low. This is possibly due to the suppressing roles of certain normal ECM components, such as Col III, biglycan, and tenascin C, on abnormal cell growth and proliferation [108, 139]. In addition, it could be that the overall microenvironment or organization of the TMG microstructures, representing a normal ECM environment, confines the cancer cells in a more differentiated state, as supported by the data in Fig. 4 and discussed, and constrains the ability of the cells to modify the matrix in favor of their growth and movement [15, 16]. However, the normal environmental suppressions on the cancer cells may be diminished over time after the cells adapt to the microenvironment and modify the matrix. Compared to TMG, Col I and Matrigel are more in common in the induction of a Warburg effect in both primary and cancerous mammary epithelial cells, which produced lactate for their growth (Fig. 6). An immediate cause for the differences in lactate production from the cells on the different hydrogels could be the distinct cell proliferation rates on the matrices [40]. It is plausible that the innate growth factors, tumor-derived molecules, and underdefined cellular proteins in Matrigel potentiated the cell proliferation and subsequent production of lactate. Future studies comparing the cell growth and metabolic phenotypes in breast tumor-derived ECM hydrogel and Matrigel cultures will provide insights into disease- and tissue-specific matrix regulation of tumor metabolism and progression.

Our NMR data collectively deliver three important messages. First, mammary epithelial cells consume more glucose and generate more lactate on Matrigel than on Col I and TMG under the same culturing conditions. Second, cancer cells produce more lactate than normal cells do on Matrigel and Col I. Third, ECM biochemical and structural properties do matter for the fate of the cells living in the matrix environment. In the absence of serum, the survival of the cancer cells seems to be totally dependent on glycolytic metabolism, where the environmental glucose is utilized by the cells to generate lactate as an energy source. This lactate source is so vital for the cells that depleting the lactate produced by the cells fully abolished their survival and proliferation in the environment favoring their growth, as exemplified by the breast cancer cells grown on Matrigel (Fig. 6). Since MM231 breast cancer cells cultured on TMG proliferated slower than they were on Matrigel [40] and secreted very less lactate to the medium (Fig. 6b), we speculate that LOX would only break down the limited amount of lactate under this situation and its inhibition of the cell proliferation on TMG would be similar to that of HUMEC on Matrigel (Fig. 6c). Future study is necessary to explore a broad inhibitory effect of LOX on cancer cell proliferation in various tumor ECM microenvironments. Targeting cancer or stromal cell production and extracellular clearance of lactate may therefore have therapeutic implications in the treatment of human solid tumors such as breast cancers.

## Conclusions

Taken together, this study highlights the distinct phenotypes and metabolic profiles of mammary epithelial cells on physicochemically different natural hydrogels. As a normal tissue-derived hydrogel with full ECM components, TMG enables the cells grown on it to exhibit certain biological properties unique to the matrix. It can serve as a tissue- and disease-specific model system to study spatial cell biology under pathophysiological conditions and provides a native-tissue mimicry tool for the identification of novel biomarkers and therapeutic targets of human cancers.

## Supplementary information

**Additional file 1: Figure S1.** MM231 cells submersed in TMG over time. The presence of the cells on the surface of polymerized TMG was observed using IF staining and fluorescence microscopy at 5-h (A), 16-h (B), 24-h (C), and more than 24-h (such as 36-h, D) time points. Col I staining, green; F-actin/phalloidin, red; nucleus/Hoechst, blue. Scale bars, 10 μm.

**Additional file 2: Figure S2.** IF staining of CD44 in MM231 cells cultured on the three types of hydrogels for more than 24 h with better cell spreading. CD44, red; Col I, green; Nucleus, blue (Hoechst staining). Scale bars, 10 μm.

**Additional file 3: Figure S3.** The morphologies of SUM1315 cells on TMG, Col I, Matrigel, and 2D glass slide at 24-h was examined using IF staining of Col I (green), F-actin/phalloidin (red), and nucleus/Hoechst (blue) coupled with fluorescence microscopy. Scale bars, 10 μm.

**Additional file 4: Figure S4.** The expression of CD44 in SUM1315 cells cultured on TMG, Col I, and Matrigel was inspected using IF staining. CD44, red; Col I, green; nucleus/Hoechst, blue. Scale bars, 10 μm.

**Additional file 5: Figure S5.** NMR spectrum of a representative medium background control sample. (A) The full spectrum of 1× RPMI 1640 medium containing 1 g/l of ^13^C_6_-labeled D-glucose that was used in the cultures of the HUMEC and MM231 cells. The spectrum of the − 3.0 – 13.0 ppm region was exhibited. (B) The vertical expansion of the view in (A) to show the background peaks derived from the culture medium per se. (C) The horizontal expansion of the 0.0–3.0 ppm region of the (A) panel where the major changes of the cell metabolites within the culture media were demonstrated in the main figures. (D) The horizontal expansion of the 3.0–6.0 ppm region of the (A) panel.

**Additional file 6: Figure S6.** NMR spectra of experimental background controls. (A) The spectra of the media from the polymerized TMG-coated culture (blue spectrum), HUMEC on TMG (red), and MM231 on TMG (green) after 1 h of incubation (37 °C, 5% CO_2_). (B) The spectra of the media from the polymerized Col I-coated culture (blue), HUMEC on Col I (red), and MM231 on Col I (green) after 1 h of incubation (37 °C, 5% CO_2_). (C) The spectra of the media from the polymerized Matrigel-coated culture (blue), HUMEC on Matrigel (red), and MM231 on Matrigel (green) after 1 h of incubation (37 °C, 5% CO_2_).

**Additional file 7: Figure S7.** NMR spectra of triplicate data sets collected from the 7-day MM231 cell culture samples. (A) The spectra of the media from the cultures of MM231 cells grown on TMG. (B) The spectra of the media from the cultures of MM231 cells grown on Col I. (C) The spectra of the media from the cultures of MM231 cells grown on Matrigel. HRMAS spectra, red = ^13^C-^1^H coupled spectra; blue = decoupled proton spectra.

## Data Availability

The datasets used and/or analyzed during the current studies are available from the corresponding author on reasonable request.

## Abbreviations

AFM: Atomic force microscopy
Col I: Collagen I (type I collagen)
DCIS: Ductal carcinoma in situ
DMEM: Dulbecco’s modification of Eagle’s medium
ECM: Extracellular matrix
EHS: Engelbreth-Holm-Swarm
FBS: Fetal bovine serum
FID: Free induction decay
GFR: Growth factor reduced
GFP: Green fluorescent protein
HRMAS: High-resolution magic angle spinning
HUMEC: Human mammary epithelial cells
IDC: Invasive ductal carcinoma
IF: Immunofluorescence
LrECM: Laminin-rich ECM (Matrigel)
LOX: Lactate oxidase
MM231: MDA-MB-231 cells
MM468: MDA-MB-468 cells
NMR: Nuclear magnetic resonance
SEM: Scanning electron microscopy
TACS: Tumor-associated collagen signatures
TMG: Tissue matrix gel
TMSP: Trimethylsilylpropanoic acid sodium salt
