# Supplementary material for: Distinct phenotypes of cancer cells on tissue matrix gel
Source: Breast Cancer Res. 2020 Jul 31;22:82. doi: 10.1186/s13058-020-01321-7 (PMC7395363; doi:10.1186/s13058-020-01321-7)
Supplement: Supplementary file 1 [file 13058_2020_1321_MOESM1_ESM.docx]

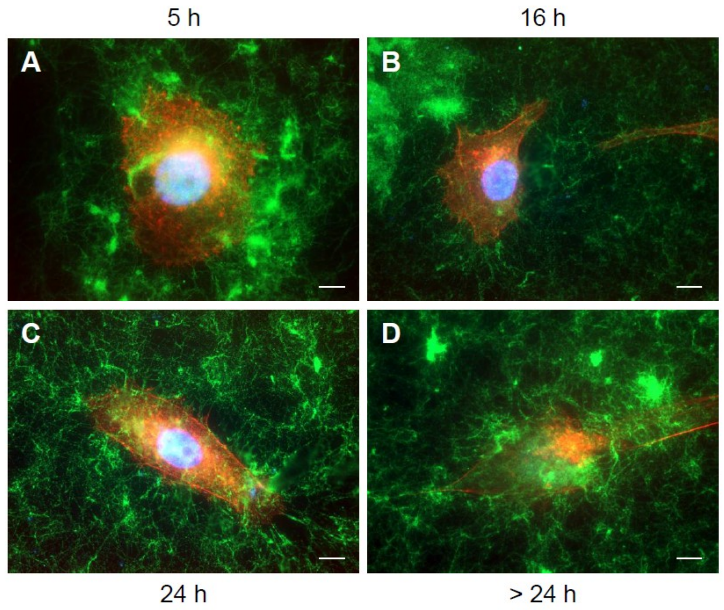
5 h
16 h
A
B
C
D
24 h
> 24 h
