# Supplementary material for: Distinct phenotypes of cancer cells on tissue matrix gel
Source: Breast Cancer Res. 2020 Jul 31;22:82. doi: 10.1186/s13058-020-01321-7 (PMC7395363; doi:10.1186/s13058-020-01321-7)

Figure S2

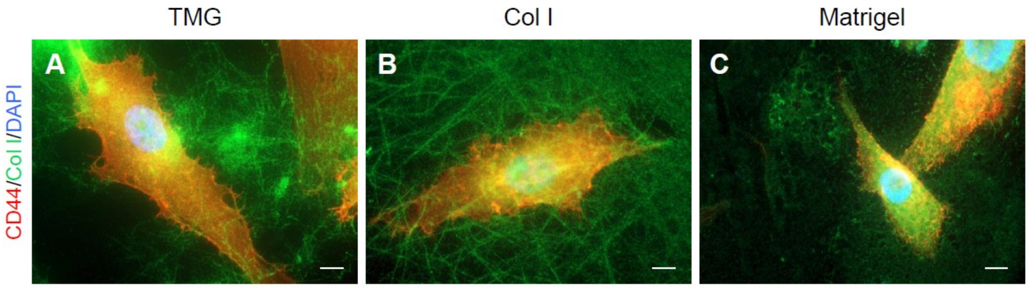

Supplement: Supplementary file 2 [file 13058_2020_1321_MOESM2_ESM.docx]
