# Supplementary material for: Distinct phenotypes of cancer cells on tissue matrix gel
Source: Breast Cancer Res. 2020 Jul 31;22:82. doi: 10.1186/s13058-020-01321-7 (PMC7395363; doi:10.1186/s13058-020-01321-7)
Supplement: Supplementary file 5 [file 13058_2020_1321_MOESM5_ESM.docx]

Figure S5

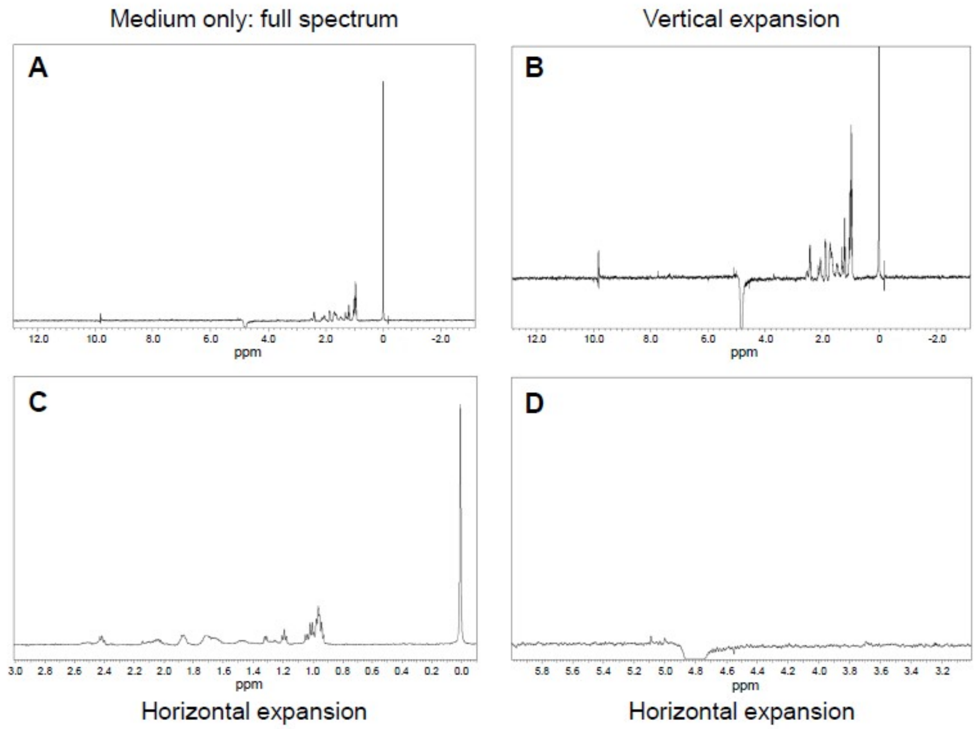

Medium only: full spectrum
Vertical expansion
A
B
C
D
12.0 10.0 8.0 6.0 4.0 2.0 0 -2.0
ppm
3.0 2.8 2.6 2.4 2.2 2.0 1.8 1.6 1.4 1.2 1.0 0.8 0.6 0.4 0.2 0.0
5.8 5.6 5.4 5.2 5.0 4.8 4.6 4.4 4.2 4.0 3.8 3.6 3.4 3.2
Horizontal expansion
Horizontal expansion
