# Supplementary material for: Distinct phenotypes of cancer cells on tissue matrix gel
Source: Breast Cancer Res. 2020 Jul 31;22:82. doi: 10.1186/s13058-020-01321-7 (PMC7395363; doi:10.1186/s13058-020-01321-7)
Supplement: Supplementary file 6 [file 13058_2020_1321_MOESM6_ESM.docx]

Figure S6

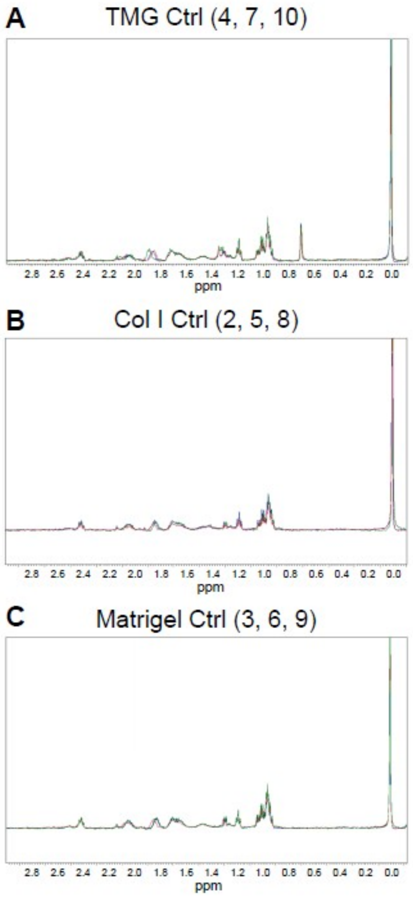
A
TMG Ctrl (4, 7, 10)
2.8 2.6 2.4 2.2 2.0 1.8 1.6 1.4 1.2 1.0 0.8 0.6 0.4 0.2 0.0
ppm
B
Col I Ctrl (2, 5, 8)
2.8 2.6 2.4 2.2 2.0 1.8 1.6 1.4 1.2 1.0 0.8 0.6 0.4 0.2 0.0
ppm
C
Matrigel Ctrl (3, 6, 9)
2.8 2.6 2.4 2.2 2.0 1.8 1.6 1.4 1.2 1.0 0.8 0.6 0.4 0.2 0.0
ppm
