# Supplementary material for: Distinct phenotypes of cancer cells on tissue matrix gel
Source: Breast Cancer Res. 2020 Jul 31;22:82. doi: 10.1186/s13058-020-01321-7 (PMC7395363; doi:10.1186/s13058-020-01321-7)
Supplement: Supplementary file 7 [file 13058_2020_1321_MOESM7_ESM.docx]

Figure S7

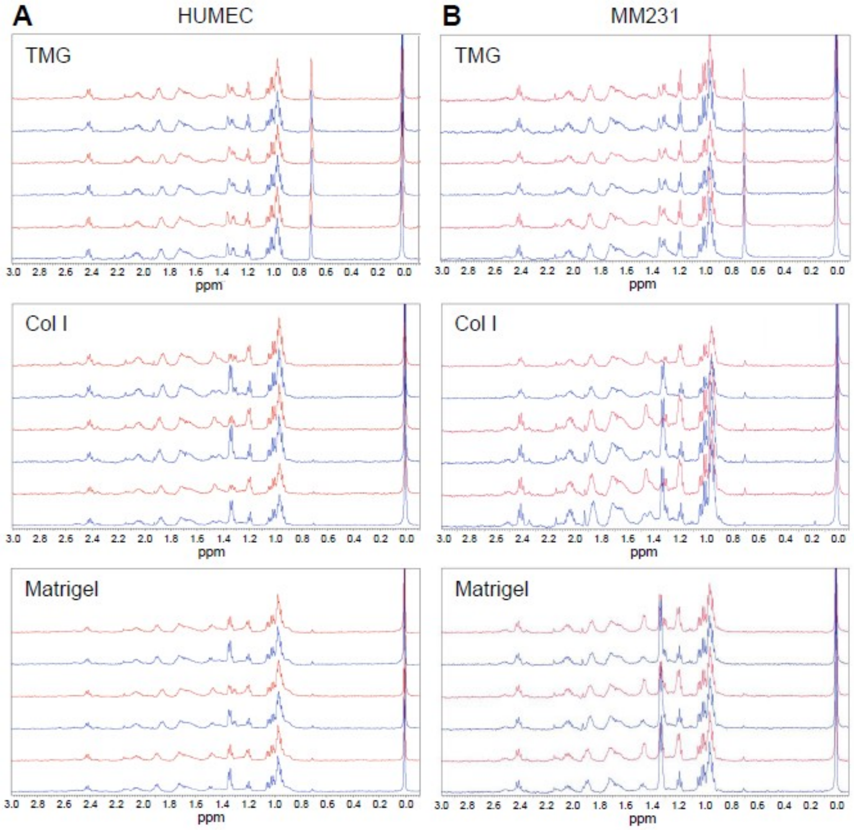

A
HUMEC
B
MM231
TMG
Col I
Matrigel
3.0 2.8 2.6 2.4 2.2 2.0 1.8 1.6 1.4 1.2 1.0 0.8 0.6 0.4 0.2 0.0
ppm
